# Crossing of the epithelial barriers by *Bacillus anthracis*: the Known and the Unknown

**DOI:** 10.3389/fmicb.2015.01122

**Published:** 2015-10-09

**Authors:** Pierre L. Goossens, Jean-Nicolas Tournier

**Affiliations:** ^1^Pathogénie des Toxi-Infections Bactériennes, Institut Pasteur, Paris, France; ^2^Unité Interactions Hôte-Agents Pathogènes, Institut de Recherche Biomédicale des Armées, Brétigny-sur-Orge, France; ^3^Ecole du Val-de-Grâce, Paris, France

**Keywords:** anthrax, macrophages, dendritic cells, epithelial cells, lethal toxin, edema toxin, spores, bacterial infections

## Abstract

Anthrax, caused by *Bacillus anthracis*, a Gram-positive spore-forming bacterium, is initiated by the entry of spores into the host body. There are three types of human infection: cutaneous, inhalational, and gastrointestinal. For each form, *B. anthracis* spores need to cross the cutaneous, respiratory or digestive epithelial barriers, respectively, as a first obligate step to establish infection. Anthrax is a toxi-infection: an association of toxemia and rapidly spreading infection progressing to septicemia. The pathogenicity of *Bacillus anthracis* mainly depends on two toxins and a capsule. The capsule protects bacilli from the immune system, thus promoting systemic dissemination. The toxins alter host cell signaling, thereby paralyzing the immune response of the host and perturbing the endocrine and endothelial systems. In this review, we will mainly focus on the events and mechanisms leading to crossing of the respiratory epithelial barrier, as the majority of studies have addressed inhalational infection. We will discuss the critical gaps of knowledge that need to be addressed to gain a comprehensive view of the initial steps of inhalational anthrax. We will then discuss the few data available on *B. anthracis* crossing the cutaneous and digestive epithelia.

## Introduction

*Bacillus anthracis*, the etiological agent of anthrax, is a Gram-positive, spore-forming bacillus. Dormant spores are highly resistant to adverse environmental conditions and they are able to survive for long periods in contaminated soils (Mock and Fouet, 2001). Anthrax is primarily a disease of herbivores, but all mammals, including humans, are susceptible. The disease is initiated by the entry of spores into the host body. This can occur via a minor lesion (cut, abrasion, fly-bite), or by eating contaminated meat or inhaling airborne spores. There are three classical types of human infection: cutaneous, gastrointestinal, and inhalational (Mock and Fouet, 2001). A recent fourth form named “injectional anthrax” has been described after an outbreak in Northern Europe caused by tainted heroin batches (Hicks et al., 2012). This later form with subcutaneous soft tissue edema without the pathognomonic black eschar of cutaneous anthrax suggests that “classical cutaneous” and injectional forms have very different pathogenesis. Each form can progress to fatal systemic anthrax. For each natural form (but not for the injectional), *B. anthracis* spores need to cross the cutaneous, digestive or respiratory epithelial barriers, respectively, as a first obligate step.

Anthrax is a toxi-infection: an association of toxemia and rapidly spreading infection progressing to septicemia. The pathogenicity of *B. anthracis* mainly depends on two plasmid-encoded major virulence factors: toxins and a poly-γ-d-glutamate capsule (PDGA), anchored to the cell wall (Candela and Fouet, 2005) which protects bacilli from the immune system, thus promoting systemic dissemination (Candela and Fouet, 2006). A more complex picture for the capsule functions has emerged beyond the simplistic view of a rampart against degradation. First, it not only protects, but also plays a role of adhesin, mediating interactions of the bacteria with the vascular endothelium, especially in the liver (Piris-Gimenez et al., 2009). Second, the pathogen sheds capsule degradation products through capsule depolymerase CapD, which are associated with virulence (Makino et al., 2002). Third, the capsule is not neutral to immune cells, as it has suppressive effects on human monocyte-derived dendritic cell (DC) functions (Jelacic et al., 2014), while another study showed that it could induce IL-1β production through caspase 1 activation on human monocyte-derived DCs (Cho et al., 2010). Interestingly, PDGA capsule of *Bacillus licheniformis* as a surrogate of *B. anthracis* capsule is a TLR2 agonist (Jeon et al., 2015), suggesting that the capsule also can alternatively activate and/or dampen the immune cells. The toxins belong to the A-B family of toxins and are composed of three proteins: edema factor (EF), lethal factor (LF), and protective antigen (PA; Mock and Fouet, 2001; Moayeri and Leppla, 2009). PA is the receptor-binding component and, after heptamerisation, can accomodate up to three molecules of EF and/or LF. The PA heptamer mediates the entry of EF and LF into the target cells and their translocation into the cytosol where they exert their toxic activities. The names edema toxin (ET) and lethal toxin (LT) designate the combination of PA with EF alone or LF alone, respectively. EF is a calmodulin-dependent adenylate cyclase that increases the intracellular concentration of cyclic AMP (cAMP). LF is a zinc-binding metalloprotease that cleaves mitogen-activated protein kinase (MAPK) kinases. These toxins alter host cell signaling, thereby paralyzing the immune response of the host and perturbing the endocrine and endothelial systems (Tournier et al., 2007, 2009b; Moayeri and Leppla, 2009). Moreover, LF also cleaves and activates NLRP1, another signaling module in certain inbred rodents, but not human (Hellmich et al., 2012; Levinsohn et al., 2012; Chavarria-Smith and Vance, 2015). It is now well established that both toxins play a critical role at two different stages of the infection: early in the infection to paralyze the immune system, and at a late stage to finally kill the host (see reviews, by Guichard et al., 2012; Liu et al., 2014). It has been shown that at the late stage of infection both toxins differentially target two vitals organs: ET-induced mortality occurs mainly through hepatocyte dysfunction, while LT induces lethality by targeting cardiomyocytes and smooth muscle cells (Liu et al., 2013). In this review we will focus on the toxin effects on the early time course of infection.

Spores germinate and establish infections at the initial site of inoculation in inhalational, cutaneous and gastrointestinal infections (nasal-associated lymphoid tissues, skin and Peyer’s patches respectively) without needing to be transported to the draining lymph nodes (Glomski et al., 2007c, 2008). Spore entry also occurs through the alveolar space, germination taking place either en route to or in the mediastinal/thoracic lymph nodes in the first steps of infection, as primitively considered (Ross, 1957; Cote et al., 2004, 2006). Thus, crossing the respiratory epithelial barrier may occur at different levels in the respiratory tract. It should be emphasized that, when a lesion of the epithelium exists, infection can initiate at the immediate site of the lesion (Ross, 1957; Gleiser, 1967; Glomski et al., 2007c). All routes of infection with encapsulated strains progress first to the draining lymph node, then the spleen acts as a reservoir, and ultimately the lungs is colonized by hematogenic route, leading to death (Glomski et al., 2007c). Absence of capsule markedly modifies dissemination patterns, the bacteria being initially confined to the portal of entry for a long period, then reaching specific organs (kidneys, gastrointestinal tract) with minimal colonization of the spleen and, late in the infection, to the lungs (Glomski et al., 2007a, 2008).

**FIGURE 1:**
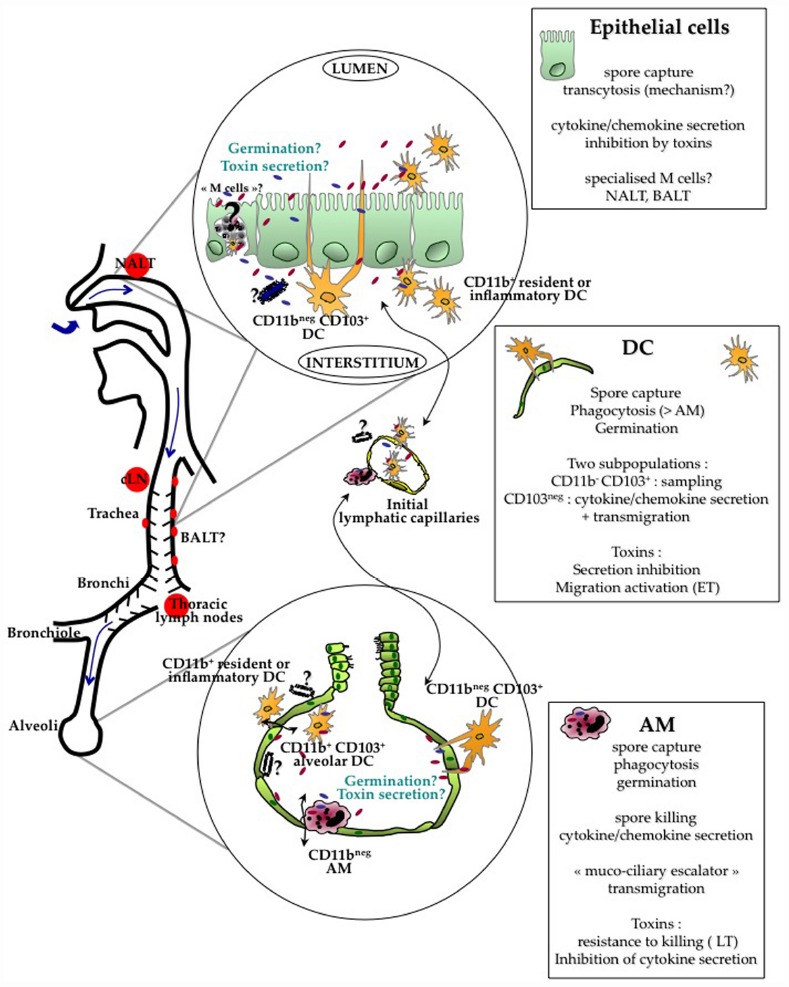
**Current evidence indicates that crossing of the respiratory barrier by the spores occurs all along the respiratory tract, from the nasopharynx to the alveoli.** The type of cells involved in the crossing is probably different at these various locations. *B. anthracis* spores cross the respiratory epithelium through the help of different lung cells : alveolar macrophages (AM), dendritic cells (DC) and/or epithelial cells. Current data suggest that AM are able to relatively control and kill the bacteria. Intraepithelial CD11b^neg^CD103^+^ DC capture the spores through cytoplasmic extension sampling the respiratory tract lumen. Epithelial cells have been shown to internalize spores and support transcytosis through still unknown mechanisms; the involvement of specialized epithelial M cells located in the epithelium overlying the lymphoid follicles over the NALT and BALT is still unknown. Spore interactions with all these cells lead to cytokine/chemokine secretion. Monocytes, DC and neutrophils are rapidly recruited in the alveolar lumen, contributing to local cytokine secretion and epithelial transmigration. Evidence suggests that germination occurs at a limited level in the respiratory tract. In simplified cellular models using AM, DC and epithelial cells, toxins globally inhibit the innate response. A key point still to be clarified is whether toxin secretion, either on the apical or the basolateral side of the epithelium positively affects epithelium crossing and further dissemination. Relative distribution of the toxin receptors on both sides is still largely unknown as well the number of spores that are required to cross the epithelium to establish a successful infection. A very low efficiency in epithelium crossing might be balanced by a high survival rate after the crossing steps. After crossing, DC and AM enter the button-like ends of the initial lymphatic capillaries and migrate into the draining lymph nodes, cervical or thoracic lymph nodes depending on the site of entry. If encapsulated bacilli or spores reach the basolateral side, migration as free bacteria into the lymphatics may also occur due to the Starling forces present in the interstitial tissue.

In this review, as the majority of studies have addressed inhalational infection, we will focus mainly on the crossing of the respiratory epithelial barrier (Figure 1); subsequent events have been considered elsewhere (Goossens, 2009). We will then discuss the few data available on *B. anthracis* crossing the cutaneous and digestive epithelia.

## Crossing the Respiratory Epithelial Barrier

As will be apparent, relatively limited data are available on the interactions of *B. anthracis* with lung-derived cells, the majority of studies having been performed with cells originating from other sources (such as blood, spleen, bone marrow, non-lung cell lines).

This section is divided in two parts. We will first examine the available data on binding, capture of the spores and subsequent immune cell activation. Then, the data on subversion of the immune response by the toxins will be detailed separately. It is in fact still unclear whether toxins affect crossing the epithelial barrier, though some evidence indicates that they might. We will conclude by considerations upon the critical gaps of knowledge that will need to be addressed to gain a comprehensive view of the initial steps of inhalational anthrax, and envision more targeted therapeutics.

### Spore Capture and Immune Cell Activation

*Bacillus anthracis* enters the host as spores. Spores are a dormant form of the bacteria. They do not present any detectable metabolic activity. They thus depend on host signals to trigger germination. During germination, spores rehydrate, resume metabolic activity, shed the external crystalline layers forming the coat and exosporium, and differentiate into bacilli, the active form of the bacteria. Bacilli produce the virulence factors that enable successful colonization of the infected host. As spores are dormant, they cannot actively cross the epithelial barrier and thus need to be captured by host cells. However, some reports have shown that *B. anthracis* spores can undergo germination in the respiratory tract (see Site of Germination and Toxin Secretion; Ross, 1957; Glomski et al., 2008; Sanz et al., 2008). In this case, the toxins, if secreted locally in sufficient amount, may play an active role, favoring crossing of the respiratory epithelial barrier by the dormant spores.

The display of specific molecules at the spore surface represents a means to attach to, and be recognized by, the cells lining or residing in the respiratory tract. The molecules implicated are not yet known, though candidates have been suggested in models using non-lung cells. For example, BclA, the main protein of the exosporium, the outer layer of the spore, has been shown to interact with integrin CR3 (CD11b), enhancing phagocytosis by human monocyte-derived macrophages (Oliva et al., 2008). Absence of CR3 correlated with a decrease in phagocytosis and some increase in virulence during infection with a non-encapsulated strain. Deletion of BclA, however, did not modify phagocytosis, showing that other spore surface components were also involved. Other reports showed the absence of effect of BclA deletion on virulence of wild-type strains (Bozue et al., 2007; Sylvestre et al., 2008). Taken together, theses studies show the implication of CR3 (CD11b) in spore capture, but as a part of more complex mechanisms. Recognition of rhamnose residues on BclA by CD14 has been suggested to increase spore binding by non-lung macrophages in activating CR3 through a TLR2 and Phosphatidylinositol 3-kinase (PI3K) process (Oliva et al., 2009). TLR on splenocytes have also been shown to recognize spores in a redundant manner, as knockout of different TLR—2, 4, and 9—did not block activation of the intracellular signaling pathways (Glomski et al., 2007b). The molecular pattern recognized are still undefined. To date, no data are as yet available on the potential critical molecules and ligands involved in the specific interactions between spore and cells of the respiratory tract. Adhesion of *B. anthracis* to the respiratory epithelium might be more complex than tested in simplified *in vitro* cell models, as the epithelial lining is not usually directly accessible *in vivo*. It is covered by an extracellular matrix, mucus or surfactant secreted by the cells. Interactions between *B. anthracis* may thus also occur indirectly through binding of the bacterial surface to these extracellular cell components, and the latter to cell receptors (for an example, see Kishore et al., 2006).

Two possibilities exist for crossing the epithelial barrier, depending on its integrity: either a lesion is present, or the integrity of the epithelium is conserved. These two hypotheses have been discussed as the “jailbreak” or “Trojan horse” models of dissemination (Weiner and Glomski, 2012). If the integrity of the epithelium is altered, spores are directly in contact with the internal milieu and the extracellular fluids, and do not necessarily need to be captured to cross the epithelial barrier. Germination is triggered *in situ*, probably through the presence of germinants at this location, which could originate from the inflammatory response and danger signals produced in response to the aggression. Bacterial growth and expression of virulence factors (capsule and toxins) then occur, enabling the bacteria to survive and colonize the host locally at the site of entry, and then to migrate via the lymphatics to the draining lymph node, before entering the blood through the thoracic duct and disseminating systemically (Glomski et al., 2007c). Indeed, lesions provoked in the larynx (or in the esophagus for gastro-intestinal infection) induce direct local infection (Glomski et al., 2007c).

If the integrity of the epithelium is not altered, capture of the spores is a crucial and obligate step in crossing the epithelial barrier and establishing a successful infection. Different cell types have been implicated in this phenomenon: alveolar macrophages (AM), DC, and, more recently, the epithelial cells lining the respiratory tract.

#### Alveolar Macrophages

Alveolar macrophages are a population of resident macrophages that are present in the alveolar spaces (Hussell and Bell, 2014). They originate from yolk sac and fetal liver progenitors during embryonic development and can be maintained throughout the adult life in the absence of monocyte recruitment (Guilliams et al., 2013; Hashimoto et al., 2013; Yona et al., 2013; Gomez Perdiguero et al., 2015). Through their phagocytic and microbicidal activities, they play a central role in clearing inert particles and micro-organisms which have passed through the defenses of the upper respiratory tract and reached the alveolar spaces.

In the 1950s, Ross (1957), in her seminal work in the inhalational anthrax model in the guinea pig, demonstrated the ability of AM to phagocytose spores in the alveolar spaces. It was an extremely rapid phenomenon, as most of the spores were phagocytosed in 35 min. We observed similar kinetics, with ∼65% of the AMs positive for spores in 10 min after murine inhalational infection (Cleret et al., 2007). Spore phagocytosis has been observed with AM from different species: mouse, guinea pig, non-human primate (NHP) and human. Interestingly, AM are CD11b^neg^ or ^low^ in mouse (GeurtsvanKessel and Lambrecht, 2008); this suggests that CR3 (CD11b) may not play a significant role in spore capture by these cells in this rodent model (cf see Spore Capture and Immune Cell Activation, Oliva et al., 2008).

*Bacillus anthracis* stimulate guinea pig AM to secrete secretory phospholipase A2 (sPLA2-IIA), a highly efficient endogenous antibiotic, CXCL-8 and the inflammatory mediator prostaglandin PGE2 (Raymond et al., 2007, 2009, 2010). Part of the observed sPLA2-IIA secretion was induced by *B. anthracis* peptidoglycan (Raymond et al., 2007). Upon phagocytosis, NHP AMs secrete proinflammatory cytokines, such as TNFα, IL1β, IL-6, and CXCL-8 (Ribot et al., 2006). Human AM stimulated with spores secrete TNFα, MCP-1, IL-1α and β, CCL3, CCL4, and IL-6 through MAPK activation (Chakrabarty et al., 2006; Wu et al., 2009).

Evidence for germination of *B. anthracis* within murine AM (Guidi-Rontani et al., 1999) confirmed *in situ* observations in the guinea pig (Ross, 1957). As spore-containing AMs were found in the tracheobronchial lymph nodes, these data were interpreted as indicating that the AM crossed the epithelial barrier to reach the extracellular space, enter the initial lymphatic capillaries and migrate into the draining lymph node, thus participating in bacterial dissemination (Ross, 1957). This interpretation is still considered valid, though evidence is currently accumulating that AM may play a different role and other cells may be involved in epithelium crossing (see Dendritic Cells and Epithelial Cells). In this respect, a key observation about the role of AM in establishing infection is that a significant proportion of the spores found in the AMs were killed (Ross, 1957). Furthermore, using macrophage depletion *in vivo* in a mouse model of inhalational infection, Cote et al. (2004) provided evidence that AMs played an important role in limiting or clearing *B. anthracis* infection. Taken together, these reports and the data on their relative resistance to LT (see Alveolar Macrophages) suggest that AM are an efficient first line of defense, able to capture spores and contain bacilli outgrowth.

Along with the resident AM population, recruitment of monocytes in the alveolar space occurs rapidly—the size of the population increases as soon as 6 h after instillation in a murine model (Cleret et al., 2007). These cells may react differently from AM to the interaction with the spores and to the toxins and control the initial steps of the infection less efficiently. We have recently shown that rapidly after infection, AMs make contact with CX3CR1-positive DCs and monocytes in the lung, suggesting that some information can be transferred through these contacts (Fiole et al., 2014). Interestingly, we have shown that NK cells activated by macrophages infected by formaldehyde-inactivated spores induces the production of IFN-γ that participate in the control of the infection in a subcutaneous model of infection (Klezovich-Benard et al., 2012). Although, it is not known if this hold true in the lung, this may be probable as 10% of lung lymphocytes are NK cells (Lysakova-Devine and O’Farrelly, 2014), this may represent another connection for AM networking.

Interestingly, what is now considered is not the crossing of the epithelial barrier by the bacteria, but by the AM (or DC, see Dendritic Cells) carrying the bacteria. The mechanisms involved are thus those of eukaryotic cell–cell interactions. The mechanisms of trans-epithelial migration (paracellular versus transcellular) of the AM, or more generally of leukocytes, are still under debate (Muller, 2003; Carman, 2009). This point is rarely addressed in the anthrax field, though it is pivotal to the understanding of the pathophysiology of inhalational anthrax.

#### Dendritic Cells

Dissemination from the lung was still observed in macrophage-depleted mice, suggesting the existence of a macrophage-independent route of capture and dissemination (Cote et al., 2004). Among the cells that could play a pivotal role are the DCs. Different DC subsets are present at all levels in the respiratory tract, exerting different functions involved in immunosurveillance (De Heer et al., 2005; GeurtsvanKessel and Lambrecht, 2008; Lambrecht and Hammad, 2012). Conventional DCs (cDCs) express high level of CD11c, compared with plasmacytoid DCs (pDCs). Little is known about the role of pDCs in anthrax infection, and we will focus this review exclusively on cDCs, although we cannot exclude that pDC subset could play a role. Schematically, the trachea and large conducting airways present a well-developed network of CD11b- CD103^+^ intra-epithelial DC that extend cytoplasmic processes between epithelial cells directly into the airway lumen; they perform sampling of the airway luminal surface. They can capture antigens, with an increased sampling after bacterial stimulation (Jahnsen et al., 2006). This phenomenon is similar to what has been reported for the capture of *Salmonella typhimurium* by intestinal DCs along Peyer’s patches (Rescigno et al., 2001). Resident and inflammatory CD11b^+^ DCs are present in the submucosa of the conducting airways and play an inflammatory role in secreting chemokines and cytokines. CD11b^+^ DCs are also found in the alveolar lumen. After sampling the environment or crossing the epithelium, lung DC traffic between the lung and the thoracic lymph nodes, alerting the innate defenses in case of danger and driving the pulmonary immune T response (Jakubzick et al., 2006).

Few studies have addressed the role of lung DCs in *B. anthracis* infection, most probably due to their low number and difficulty of isolation. Most available data on DC have been obtained mainly on non-lung DC, murine or human DC isolated from Flt3-treated spleen or expanded from monocyte or bone marrow *in vitro* with growth factors (Agrawal et al., 2003; Brittingham et al., 2005; Tournier et al., 2005). Human monocyte-derived DC phagocytose spores predominantly by coiling phagocytosis *in vitro* (Brittingham et al., 2005). This type of phagocytosis is observed with other pathogens such as *Legionella pneumophila*, *Borrelia burgdorferi*, spirochetes, *Francisella tularensis*, and trypanosomatids (Rittig et al., 1998). Spore infection in this model of non-lung DC triggered a loss of tissue-retaining chemokine receptors and an increase in lymph node homing receptors; the inference is that migration of the infected DC to the draining lymph nodes would be increased, thus favoring dissemination of the pathogen (Brittingham et al., 2005). It must be stressed that translating results from non-lung DC to lung DC cannot readily be performed as very different cytokine secretion patterns, for example, were found for lung DC and BMDC (Cleret et al., 2006).

We showed that murine lung DC phagocytosis spores more efficiently than AMs *in vitro* and *in vivo* (Cleret et al., 2006). Upon phagocytosis, they secrete cytokines and chemokines, such as TNFα, IL-10, and IL-6. In a further study, we addressed *in vivo* the role of lung DC in spore uptake and dissemination (Cleret et al., 2007). Using fluorescent spores and CX_3_CR1^+/gfp^ mice that specifically express GFP in DC, we have shown *in vivo* phagocytosis of fluorescent spores by the GFP-lung DCs. The lung DCs were able to sample the spores through the respiratory epithelium without crossing the epithelial barrier, by emitting cytoplasmic pseudopods that cross the epithelial barrier, sample the alveolar space and capture the spores (Cleret et al., 2007). By dynamic imaging, we have shown on lung tissue explants that the capture of a spore is a rapid phenomenon (less than 3 min; Fiole et al., 2012). Through this mechanism, the spores cross the epithelial barrier.

Furthermore, during infection, recruitment of DC into the alveolar space was observed as early as 6 h after instillation, and 80% of them had phagocytosed spores (Cleret et al., 2006). Further migration of these recruited DCs through the epithelial barrier is believed to happen in the same way as for AMs. Similar gaps of knowledge exist for the mechanisms of transmigration involved. After transmigration, the infected lung DCs reach the lung parenchyma where they enter the initial lymphatic capillaries and migrate to the draining lymph nodes.

The following picture is now emerging in which AMs capture and destroy *B. anthracis* spores, while lung DC sample and transport spores. As AMs seem more prone to kill *B. anthracis* once germination has been triggered, lung DCs—which are not considered as microbicidal effector cells—would thus seem to play a major role in promoting dissemination from the alveolar space to the draining lymph nodes.

#### Epithelial Cells

A role for lung epithelial cells has been suggested in spore capture and trans-epithelial migration. In reassessing histological data provided by Ross in the previous millennium (Ross, 1957), spores were often seen lying in close apposition to the lining of the alveolar ducts and the alveoli, and thus prone to interaction with epithelial cells. Xu and collaborators have shown that a human lung epithelial cell line (model of type II alveolar cells) and primary human small airway epithelial cells could bind and internalize *B. anthracis* spores *in vitro* (Russell et al., 2008b). Internalized spores were able to survive and translocate from the apical to the basolateral side without disrupting the barrier integrity. In an attempt to test for *in vivo* relevance of this type of cell interactions, they then resorted to the mouse model of inhalational infection, model more amenable to direct analysis (Russell et al., 2008a). They showed that spores were associated with the epithelial surfaces in the airways and alveoli and taken up by lung epithelial cells *in vivo*. A key point to be addressed now is: what are the mechanisms of spore capture and translocation in these non-phagocytic cells? Though an interesting non-exclusive alternative to AM and DC, the relative contribution of lung epithelial cells to spore entry *in vivo* remains to be ascertained, as the efficiency of spore capture seems rather low (Russell et al., 2008a,b; Tournier et al., 2009a). However, high efficiency might not be required for establishing a successful infection (see After Crossing the Epithelium Barrier).

Apart from a possible role in directly mediating crossing of the epithelial barrier, epithelial cells also participate in the innate immune response by reacting to bacterial and inflammatory stimuli and secreting cytokines and chemokines (Hammad and Lambrecht, 2008; Suzuki et al., 2008). The chemokines attract neutrophils, monocytes and DC in the airways, while cytokines induce DC maturation.

Few studies have addressed this point in inhalational anthrax, though the role of epithelial cells in other pathologies has been explored for many years. Raymond et al. (2009) have shown that epithelial cells, from either a murine or human source, when stimulated by *B. anthracis*, secrete CXCL-8/KC, IL-6, and CXCL-2. In an attempt to gain access to the potential interactions that could exist between lung cells, Chakrabarty et al. (2007) used a lung slice model of *in vitro* infection, thus retaining the complex architecture of the lung tissue. Through immunohistochemistry, they showed that exposure to *B. anthracis* spores induced production of IL-6 and CXCL-8 by alveolar epithelial cells and macrophages.

Mucosa-associated lymphoid tissue (MALT) lies beneath the epithelium of many mucosal surfaces. Microfold (M) cells are specialized epithelial cells that actively capture and transport soluble and particulate compounds across the epithelial barrier. They have been extensively studied and described in Peyer’s patch in the gut. The presence of a specialized epithelial cell type with similar functions has been observed in the respiratory tract in the rabbit and the mouse (Richmond et al., 1993; Gebert and Pabst, 1999; Tango et al., 2000). Their relative importance in spore capture awaits further analysis.

Clearly, owing to the paucity of data on the role of lung epithelial cells in inhalational anthrax, further studies should focus on this cell type to ascertain its contribution in infection control, and its subversion by *B. anthracis* virulence factors.

#### Other Cell Types

***Neutrophils***

Among other cell types that could be implicated in the response to *B. anthracis* inhalational infection, few studies have addressed the potential role of neutrophils. Neutrophils are highly microbicidal cells that belong to the first wave of cell recruitment in an infected site and participate in host innate defenses and adaptive immunity (Appelberg, 2007; Yang et al., 2009). Furthermore, neutrophils have been shown to transport pathogens in cutaneous models of infection (Abadie et al., 2005; Appelberg, 2007). Neutrophils are recruited by chemokine CXCL-8/KC gradients. As described above, AMs (Ribot et al., 2006), lung epithelial cells (Raymond et al., 2009) and DCs (Brittingham et al., 2005; Cleret-Buhot et al., 2012) secrete this neutrophil-recruiting chemokines. In a guinea pig model of inhalational infection, recruitment of neutrophils was observed at least 16 h after infection (Raymond et al., 2009), and even 6 h after murine inhalational infection (Sanz et al., 2008). Finally, neutrophils have been shown to play a role in host defense in pulmonary anthrax along with AMs, as *in vivo* neutrophil depletion increased mortality in a mouse model (Cote et al., 2006; Garraud et al., 2012). We have identified a subpopulation of neutrophils recruited in the lung producing IL-17A that were instrumental to the self-recruitment of this population depending on the IL-17A/F axis (Garraud et al., 2012). These cells may thus play an indirect role in hindering successful crossing of the epithelial barrier, by clearing the bacteria *in situ* or increasing the inflammatory response and inflammatory monocyte and DC recruitment. On the other hand, one may speculate that an excessive neutrophil response may induce damage to the epithelial barrier, thus favoring bacterial entry (Message and Johnston, 2004). Such potential immuno-pathologic effects await further studies.

***Patrolling monocytes***

Peripheral monocytes are a heterogeneous population of two subsets: on one hand “classical” monocytes (CX3CR1^low^Ly6C^high^) also referred to “patrolling” monocytes thought to patrol and maintain endothelial cells; and on the other hand “non-classical” monocytes (CX3CR1^high^,Ly6C^low^), sometimes referred to as “inflammatory” monocytes, as they can serve of precursor of macrophages and DCs under inflammatory conditions (Geissmann et al., 2003, 2010). Recent, studies have shown that Ly6C^+^ monocytes constitute a precursor of Ly6C-negative blood resident monocytes (Yona et al., 2013). Recent studies have shown that Ly6C^+^ monocytes constitute a population of resident monocytes in the lung that can patrol, capture antigen to carry up to the lymph node in absence of inflammatory stimulus (Jakubzick et al., 2013). Another study has shown that lung monocytes patrol lung alveoli and lung capillaries by locating specifically at the interface between lung capillaries and alveoli (Rodero et al., 2015). The role of monocytes patrolling lungs and transporting antigen to the lymph nodes, while established at the steady state, needs to be examined in an infectious context. It may play an important role to clear the alveoli, and transport antigen to the draining lymph nodes.

### Subversion of the Lung Innate Immune Response by *B. anthracis* Toxins

Crossing the epithelial barrier is not sufficient for *B. anthracis* to establish a successful infection and to colonize its host. It needs to subvert the local innate immune response.

It is widely considered that the capsule plays a role at a later step of infection, after epithelium crossing, when the encapsulated bacilli reach the extracellular milieu, by blocking phagocytosis and protecting the bacteria from the bactericidal innate immune effectors. However, the initial time points when, and tissue locations where, encapsulated bacilli are released are still largely unknown. Could toxin secreting encapsulated bacilli be found, even in small numbers in specific locations, on the aerial side of the respiratory tract? Could spores be released on the basolateral side of the respiratory epithelial lining, differentiate into toxin-secreting encapsulated bacilli and affect epithelial cell viability and function from “within”? Both scenarii could modify the integrity of the epithelial barrier and increase further bacterial crossing. Finally, the pseudo-proteic capsule enables adhesion of *B. anthracis* to the vascular endothelium, especially in the liver (Piris-Gimenez et al., 2009) and play a role in virulence to be better studied (Makino et al., 2002). Such interactions may influence local adhesion and further migration.

Subversion of the lung innate response is achieved primarily through the action of *B. anthracis* LT and ET that paralyze the immune system (Moayeri and Leppla, 2009; Tournier et al., 2009b). Secretion of the toxins is considered an early event after germination. PA mRNA is detected as early as 15 min after triggering of germination (Cote et al., 2005). Protein expression studies (fusion with β–Galactosidase) showed that LF and the toxin trans-activator AtxA were detectable within 3 h of macrophage infection (Guidi-Rontani et al., 1999). Taken together, and provided that a sufficient level of germination is achieved in the respiratory tract, the toxins may intervene in favoring the crossing of the epithelial barrier. To note, as mentioned above, the effects of the toxins have rarely been measured on lung resident cells.

Another key point is the local toxin concentration in the infected epithelia at this early stage of infection. The only currently available data are the serum levels at the terminal stage of infection (10–35 *μ*g/ml) in rabbits (Mabry et al., 2006; Molin et al., 2008). Determination of the toxin levels in the epithelia at the early stage of infection needs highly sensitive assays; such assays have been recently described for EF (Duriez et al., 2009) and LF (Boyer et al., 2007) and should answer this key point in subversion. Preliminary studies focused on LT level measurement by coupling immune-precipitation and mass-spectrometry in cutaneous model of infection in mice (Weiner et al., 2014) and in an aerosol model of infection in NHP (Boyer et al., 2009). The rodent model data showed that LT was detectable at an early stage of infection locally and in the blood (Weiner et al., 2014). Although, those data are not translatable on a pulmonary model of infection, one may infer that cells at the port of entry may be exposed at significant level of toxins.

#### Alveolar Macrophages

The majority of the studies on the effects of the toxins on macrophages were performed on macrophage cell lines or monocyte-derived or bone marrow-derived macrophages from diverse species (Tournier et al., 2009b). Immune suppression and cell death are the most reported effects of the toxins (Moayeri and Leppla, 2009; Liu et al., 2014). Interestingly, recent studies show that ET is able to increase cell mobility in macrophages (Kim et al., 2008), suggesting an opportunity in increasing cell migration and bacterial dissemination. The inference from these studies was that AM behaved similarly. Evidence summarized below suggests a different picture.

NHP AMs are resistant to the killing effect of LT, and phagocytosis was unimpaired (Ribot et al., 2006). However, their ability to secrete proinflammatory cytokines, such as TNFα, IL1β, IL-6, and CXCL-8 was impaired and clearance of *B. anthracis* was decreased. In contrast, human AM were found resistant to LT-mediated suppression of cytokine expression. MAPKK cleavage was observed only for MEK 1 and for a high dose (5 *μ*g), probably non-physiologically encountered locally in the epithelium (or only at the late septicaemia stage of infection; Wu et al., 2009). This absence of effect of LT was correlated with a relative lack of expression of the anthrax toxin receptors, thereby leading to absence of significant binding of PA to the AM cell surface. On the other hand, this observation was species-specific, as mouse AM bound PA and their MEK-1,2,4, and 6 were cleaved. Indeed, we observed that mouse AMs were killed *in situ* in the alveoli during inhalational infection with a non-encapsulated strain (Glomski et al., 2008). Interestingly, using an *in vivo* mouse model of intra-peritoneal infection, Terra et al. (2010) showed that macrophage lysis by LT could promote an early inflammatory response leading to increased resistance to infection (Terra et al., 2010). Whether this observation could apply to AMs in the alveoli awaits further study.

Studying the effect of ET on guinea pig AM, Raymond et al. (2007) showed an inhibition of secretion of sPLA2-IIA, while CXCL-8 and PGE2 secretion was not affected. Interestingly, this effect was at the transcriptional level via a cAMP/protein kinase A-dependent and Epac-independent process. Phosphorylation of CREB was also induced by ET, but was not implicated in sPLA2-IIA transcription inhibition. Another feature of the inhibitory effect of ET was that translocation of NFkB, the transcription factor implicated in sPLA2-IIA gene transcription, was unaffected by ET. LT provoked a similar inhibition of sPLA2-IIA secretion without interfering with NF-*κ*B translocation into the nucleus (Raymond et al., 2010). This suggests that the inhibitory effects of ET and LT occur downstream of transcription factor nuclear translocation (see Epithelial Cells). Interestingly, we have shown recently that ET affects the fine regulation of actin cytoskeleton of bone marrow-derived macrophages, which would in turn disrupt main cell functions (Trescos et al., 2015).

The emerging picture is that, though differences in resistance to LT exist between species, AM seem to present a certain level of resistance to the toxic effect of LT. The primary role of toxins currently proposed would be not killing of AM, but down-regulating the AM immune response, thus facilitating bacterial survival and spreading.

#### Dendritic Cells

The majority of the studies addressing the effects of *B. anthracis* toxins on DC were performed on non-lung DC *in vitro*. Translation of theses observations to *in vivo* is still pending. Our studies are currently the only ones performed *ex vivo* and *in vivo* on lung DC (Cleret et al., 2006, 2007; Fiole et al., 2014), thus giving a hint of what actually occurs during an infection.

We will first briefly summarize the data on the interactions between *B. anthracis* and non-lung DC. *B. anthracis* toxins globally inhibit secretion of a wide range of cytokines and chemokines, such as TNFα, IL1α, IL-6, CXCL-8, IL-10 and IL-12, in DC from different sources—spleen DC from Flt3-treated mice (Agrawal et al., 2003), murine BMDC (Tournier et al., 2005; Cleret et al., 2006), human monocyte-derived DC (Brittingham et al., 2005). LT and ET inhibited most inflammatory chemokine production (CCL2, CCL3, CCL4, CCL5, CXCL-8) in human monocyte-derived DCs, but only LT inhibited neutrophil attraction in a transwell assay (Cleret-Buhot et al., 2012). Conflicting effects on maturation have been reported, either inhibition (Agrawal et al., 2003), or no effect (Cleret et al., 2006). Interestingly, ET has been found to exert a positive effect on migration of human monocyte derived DC (Maldonado-Arocho and Bradley, 2009). The inference is that ET could favor dissemination of spores once phagocytized by DC. Interestingly, this could be correlated to our study showing that ET markedly modified the patterns of bacterial dissemination in mouse and guinea pig models, leading to apparent direct dissemination to the spleen and provoking apoptosis of lymphoid cells (Dumetz et al., 2011).

The only available study on lung DC in a murine model of inhalational infection showed that an LT-secreting strain inhibited IL-6, IL-10, and TNFα secretion, whereas an ET-secreting strain inhibited only TNFα secretion, increased IL-6 secretion while IL-10 was not affected (Cleret et al., 2006). The effects observed during infection with a strain secreting both toxins showed the predominance of the LT phenotype. The emerging picture is that lung DC efficiently phagocytosis *B. anthracis* spores, the toxins paralyze the DC cytokine secretion while enhancing the ability of cells to migrate to the draining lymph nodes, thus increasing the potential for dissemination of the bacteria.

#### Epithelial Cells

There have also been a limited number of studies on the effects of the toxins on lung epithelial cells. In a murine model of inhalational infection, no macroscopic cytotoxic effects were observed in histology in the epithelium lining the alveoli, though microcolonies of LT- and ET-secreting bacteria were filling the alveolar spaces (Glomski et al., 2008). Using *ex vivo* differentiated human lung epithelium, Lehmann et al. (2009) suggested that long term incubation (24–48 h) with LT altered the actin cytoskeleton and microtubule network, leading to impairment of the epithelial barrier function (Lehmann et al., 2009), as also observed in human endothelial cells (Rolando et al., 2010). Using either a human bronchial epithelial cell line or isolated murine lung epithelial cells, Raymond et al. (2009) showed that LT induced an inhibition of secretion of CXCL-8/KC, IL-6, and MIP-2. This inhibition was acting at the nuclear level, through the downstream effect of MAPK inhibition of phosphorylation, by blocking epigenetic modifications of the chromatin (histone H3 phosphorylation), thus inhibiting binding of NF-*κ*B, key transcription factor for CXCL-8 in this cell type, to the CXCL-8 promoter. Similar inhibition of transcription, while nuclear translocation of the transcription factor was unimpaired, was observed with LT and ET on the secretion of sPLA2-IIA in guinea pig AMs (see Alveolar Macrophages, Raymond et al., 2007, 2010). Taken together, these results suggest that inhibition of epigenetic mechanisms by the toxins might be a more general way of blocking immune effector and mediator transcription. If this is the case, this might lead to more targeted therapeutics to rescue the functions of already intoxicated cells. Another study on human lung epithelial cells has shown that LT caused actin rearrangement and impaired desmosome, as well as reduced surfactant production consistent with lung barrier impairment (Langer et al., 2012).

By inhibiting cytokine secretion in response to the infectious process, the toxins will block the inflammatory reaction, thus enabling the infection to proceed silently and be detected only when it is too late to control. By paralyzing the immune response, they would also allow survival of the germinating spores, at a step when they are highly vulnerable to the host defenses.

### Considerations on Key Aspects of *B. anthracis in vivo* Life Cycle

Till recently, interpretative models of crossing of the respiratory epithelium in inhalational anthrax and subsequent events were based mainly on the fragmentary data obtained in simpler cellular models. The majority of these studies have used a reductionist approach and have focused on potential interactions with specific cell types. The valuable and highly informative data obtained demonstrate that the mechanisms observed exist, but their relative importance and actual relevance in inhalational anthrax remain undefined. There is a need to move toward more complex models, in particular to ascertain the sites of spore entry and of toxin secretion. Both aspects are key steps in understanding how *B. anthracis* cross the respiratory barrier. Some studies have recently attempted to explore these events, using new technologies, in systems more closely modeling the pathophysiology of a lung infection (Cleret et al., 2006, 2007; Chakrabarty et al., 2007; Glomski et al., 2007c, 2008; Russell et al., 2008a; Sanz et al., 2008). They were able to approach what actually happens *in vivo*, paving the way for a clarification of the initial steps of infection.

#### Portal of Entry

In the 1950–1960s, it was clearly established that the first steps of inhalational anthrax did not involve infection of the lung parenchyma, and did not represent a true primary pneumonia (Hughes et al., 1956; Ross, 1957; Lincoln et al., 1965). The alveolar space was, until recently, considered as the main portal of entry for the spores, followed by dissemination to the mediastinal/thoracic draining lymph nodes. However, due to the recent availability of powerful imaging technologies, Glomski et al. (2007c, 2008) showed, using both encapsulated and non-encapsulated *B. anthracis* in mice, that the main portal of entry occurred in the nasopharynx. *B. anthracis* were found in the NALT where they multiplied extensively, while the epithelium layer covering the lymphoid formation appeared intact by histological staining (Glomski et al., 2007c). Involvement of the nasopharynx during inhalational infection was confirmed in another study (Loving et al., 2009). Furthermore, when the nasopharynx was bypassed by direct intra-tracheal spore inoculation, infection initiated in the cervical lymph nodes, showing that the spores were captured in the upper respiratory tract and migrated to the regional draining lymph nodes (Glomski et al., 2007c). Further studies are needed to address the role of the epithelium covering the bronchus-associated lymphoid tissue (BALT) in spore capture and entry (DC, epithelial cells, M cells); these lymphoid tissues are found all along the human bronchial tract and are induced by stimulation (Richmond et al., 1993; Foo and Phipps, 2010). DC and/or epithelial cells are most probably the main cell types involved in spore capture in the upper respiratory tract. Considering the physiology of particle deposition in the airways, particles even of a small size that can reach the alveolar space, can deposit in the entire respiratory tract, in particular in the nasopharynx and along the bronchiolar epithelium. As the nose is the first filter encountered by inhaled particles, the probability for a spore to interact with the respiratory epithelium is thus higher in the nasopharynx, decreasing along the different parts of the respiratory tract down to the alveoli. Hence this will influence the probability of spore capture and epithelium crossing along the respiratory tract and, by corollary, the type of cell implicated in the crossing of the epithelial barrier. *B. anthracis* has nevertheless been detected in the mediastinal/thoracic lymph nodes in the first steps of infection (Ross, 1957; Cote et al., 2004, 2006), showing that spore entry also occurs through the alveolar space, as primitively considered. Interestingly, in the studies tracking the early stage of infection in real time through bioluminescence imaging, no bioluminescence could be detected in the mediastinal/thoracic lymph nodes; the bacterial load was at a low level and mainly in the non-germinated form (Glomski et al., 2007c, 2008; Sanz et al., 2008).

Taken together, the available data show that crossing the respiratory epithelial barrier may occur at different levels in the respiratory tract. Clearly, the cells involved (DCs, epithelial cells or AMs) and the mechanisms triggered will be different whether in the nasopharynx, along the bronchial surface or in the alveolar space. A better understanding of the relative importance of these different locations will lead to an increase in knowledge of the mechanisms involved and could lead to better targeted therapeutics.

Finally, it should be remembered that a non-exclusive additional portal of entry could be at the site of a concomitant inflammatory and infectious lesion where the integrity of the epithelial barrier is affected. Indeed, when a lesion of the respiratory epithelium exists, infection initiates at the immediate site of the lesion (Ross, 1957; Gleiser, 1967; Glomski et al., 2007c).

#### Site of Germination and Toxin Secretion

One main point of contention is the location of spore germination, and, by way of consequence, of toxin secretion, after entry into the respiratory tract. Germination is a key step for the *B. anthracis* life cycle, as it will produce its virulence factors and subvert the host innate defenses. Defining the site of germination is thus critical to understanding whether crossing the epithelial barrier is influenced by toxin production.

Significant germination in the lung tissue has rarely been reported and was usually related to inadequate sampling procedures. For example, spore germination is triggered by homogenisation and physical pressure, especially when the sample temperature increases (Jones et al., 2005; Cote et al., 2006). From bacterial bulk quantification data in lung tissue and broncho-alveolar fluids under controlled homogenization procedures, it was assumed that the respiratory lumen was not a milieu permissive for spore germination (Guidi-Rontani et al., 1999; Cote et al., 2006). Furthermore, spores can persist for many weeks in the lung tissue, necessitating prolonged antibiotic therapy (up to 60 days) to ensure absence of relapse once the treatment is stopped (Friedlander et al., 1993).

However, the status of a negative result is always ambiguous. Absence of detection of vegetative cells could also be observed if killing of the germinating spores was highly efficient. Discrimination between these two hypotheses was approached through visualization of the events occurring *in situ*. Through histologic analysis, Ross observed early germination of spores of a wild-type *B. anthracis* in the guinea pig lung aerial space (Ross, 1957). Using non-encapsulated toxin-secreting bacteria that produce bioluminescence upon germination in an immuno-deficient mouse, Sanz et al. (2008) showed rapid germination in the alveoli, as early as 30 min after infection. In the same mouse model and using non-encapsulated bacteria bioluminescent upon toxin expression, Glomski et al. (2008) showed that bacterial growth could occur as a focal point of bioluminescence in the lung tissue corresponding to micro-colonies of bacteria in the alveolar spaces. This particular mouse model (A/J strain) is very sensitive, due to the presence of a deficiency in the immune system (C5 component). Another argument discussed earlier is that ET modifies the pattern of dissemination in mouse and guinea pigs model, suggesting very early delivery of the toxins to trigger their effects (Dumetz et al., 2011). The remaining immune defenses, further impaired by the toxins secreted by the nascent bacilli, cannot control bacterial growth, leading to visualization of local alveolar infection normally controlled by adequate immune defenses. This hypothesis is further strengthened by a recent study showing that LT (but not ET) is required for full virulence in a model of pulmonary challenge in macaques (Hutt et al., 2014).

Thus, the available data show that the alveolar space and the alveolar cells are able to trigger a limited level of germination. Data gained in *in vitro* macrophage infection suggest that germination occurs during association of the spore with the macrophage, and in particular the AM, during phagocytosis (Guidi-Rontani et al., 1999). It must be remembered that germination can also be triggered at the site of an inflammatory or concomitant infectious lesion (Ross, 1957; Gleiser, 1967; Glomski et al., 2007c). The relevance of natural occurrences of such macro- or micro-lesions in germination triggering remains to be demonstrated. As they are not detected by usual techniques, the great majority of “germinating spores” are presumably rapidly killed, by lung defenses still to be identified, which may include AMs, sPLA2-IIA, defensins, lung surfactant, etc.

The corollary is that toxins may play a role in impairing the local immune response, as their expression is rapid upon germination (see Subversion of the Lung Innate Immune Response by *B. anthracis* Toxins). We have detailed above the effects of the toxins on the different cell types encountered by *B. anthracis* in the respiratory tract. Depending on the portal of entry and the first cells encountered, the effects can be different and complex. In this case, a short time window will exist during which a delicate balance between subversion by the toxins secreted by the nascent bacilli and the killing mechanisms of the resident lung cells takes place.

One critical point for the pathophysiological significance of toxin secretion in the respiratory tract, is the relative distribution of the toxin receptors on the apical vs basolateral sides of the polarized epithelial cells. The only available data on this aspect has been obtained with a human intestinal cell line (Beauregard et al., 1999). Using ET and cAMP-regulated chloride ion secretion as an index of toxin entry, the authors showed that toxin entry occurred only by the basolateral side facing the interstitium, and not the apical side facing the lumen. To our knowledge, no other reports have addressed this point, especially in a respiratory epithelium, highly relevant to the pathophysiology of inhalational anthrax. The expression of one of the two known receptors, ATR/TEM8, was determined in murine and human cutaneous, digestive and respiratory epithelia (Bonuccelli et al., 2005). The receptor was highly expressed in the epithelium of the bronchi, and particularly abundant in the ciliated epithelial cells at this location, and in the smooth muscle cells surrounding the vessels. Labeling of the epithelial cells lining the alveoli was suggested. Unfortunately, the relative labeling of the apical versus the basolateral side of the epithelial cells was not addressed. Furthermore, data on the relative distribution of the second anthrax toxin receptor, CMG2, are still lacking. Using knockout mice for each anthrax toxin receptors, Liu et al. (2009) have shown that TEM8 plays a minor role in toxin pathogenesis in mice, whereas CMG2 is the receptor mediating lethality of anthrax toxin *in vivo*.

#### After Crossing the Epithelium Barrier

Another key issue is: what happens on the other side of the epithelial barrier? Are the bacteria (spores, “germinating spores,” nascent bacilli) still inside the cells that helped them cross the barrier, or are they free on the basolateral side of these polarized cells? The classical view uses the concept of Trojan horse, or even “galloping” Trojan horse (Cleret et al., 2007), where AM and/or DC respectively enter the specialized button-ends of the initial lymphatic capillaries (Baluk et al., 2007) after crossing the epithelium and migrate into the draining lymph node; there the infection develops. Another non-exclusive possibility would be that the bacteria are released on the basolateral side through the cytotoxic effects of the toxins on the carrier cells. There, they will either be phagocytosed again, if not sufficiently protected by the antiphagocytic capsule, or migrate as free bacteria into the lymphatic vessel carried by the normal physical pressure existing in the interstitial spaces (resulting from the Starling forces in the tissues). This concept has been coined as the “jailbreak” model of infection (Weiner and Glomski, 2012). An interesting point is that, according to this possibility, the bacteria would be able to secrete toxins and impair the epithelial cell innate defenses from the basolateral side of the epithelial barrier. A consequence would be to favor further entry of bacteria from the aerial lumen.

Another unknown is the number of spores needed to successfully cross the epithelial barrier and initiate infection. A very low number could possibly be sufficient, once the bacteria has protected itself in its capsule and paralyzed the immune defenses through secretion of its toxins. If this number is low, then epithelial cells, micro-lesions of the epithelium or a few lung DCs having successfully sampled the respiratory lumen, could play a significant role in establishing disease. Recent data on a model of A/J mice infected intra-nasally by spores of an encapsulated strain has identified two independent bottlenecks depending on the port of entry (the NALTs or the lungs in this specific model of infection; Lowe et al., 2013). The founder effect identified in these studies suggests that the infection is established by a limited number of bacteria after the crossing of the epithelium.

## Crossing other Epithelia

### Crossing the Cutaneous Epithelium

It is generally considered that *B. anthracis* spores do not cross a normal cutaneous epithelium, presence of a lesion (cut, abrasion, fly-bite) is necessary (http://www.who.int/csr/resources/publications/AnthraxGuidelines2008/en/index.html). Cutaneous anthrax in humans is, in the great majority of cases, associated with a lesion. Germination is rapidly triggered upon access to the subepidermal tissue (Hahn et al., 2005; Bischof et al., 2007; Corre et al., 2013), showing the availability of germinants in the skin. In mouse infected abraded skin, invasion and proliferation of bacilli was observed within hair follicles (Watts et al., 2008). Keratinocytes have been shown to express the ATR/TEM8 receptor (Bonuccelli et al., 2005). These cells are a first line of innate defenses, as they secrete cytokines and chemokines (Tuzun et al., 2007). Recruitment of neutrophils is rapid (Cromartie et al., 1947; Hahn et al., 2008) and leads to either local control of the infection—before antibiotics and vaccine were available, cutaneous anthrax in human was controlled in 60–90% of the cases; http://www.who.int/csr/resources/publications/AnthraxGuidelines2008/en/index.html—or to extensive local bacterial multiplication and dissemination to the draining lymph nodes (Cromartie et al., 1947). In the latter case, the cutaneous lesion, with necrosis and edema, is characteristic leading to a black eschar. In fact, there is no good animal model of natural human cutaneous anthrax, as most of laboratory models uses sub-cutaneous or at best intra-dermal injection in the ear pinna, which resembles more to the novel “injectional” form in human leading to soft tissue infection. New delivery devices from nanotechnology used for skin immunization such as microneedles may help to develop a real animal model of cutaneous anthrax (Kim et al., 2012).

### Crossing the Digestive Epithelium

In contrast to inhalational anthrax, the model of gastro-intestinal (GI) anthrax has not been developed extensively, and *B. anthracis* crossing of the digestive epithelium has rarely been analyzed, although it should be kept in mind that anthrax is the archetype zoonosis and it represent its main natural form of the disease in the livestock (Beyer and Turnbull, 2009). The role of potential lesions in the predisposition to GI anthrax, comes from the observation that anthrax outbreaks tend to occur after droughts that increases abrasions by dried plants, although many other ecological and environmental factors may be involved (Van Ness, 1971; Beyer and Turnbull, 2009). Russell et al. (2007) have shown that *in vitro* spores can bind and be internalized by the intestinal cell line Caco-2. Anthrolysin O has also been shown to be able to disrupt Caco-2 cell epithelium function and open the passage for vegetative bacteria (Bishop et al., 2010). More interestingly, following in real time the dynamics of GI infection through bioluminescent imaging, Glomski et al. (2007c) have shown that spore capture and proliferation occurred at Peyer’s patches. As mentioned above, DC and/or the specialized epithelial M cells may be the cells involved. If a lesion of the epithelium was present, infection initiated at the site of the lesion (Glomski et al., 2007c). The ATR/TEM8 toxin receptor is expressed on the epithelium of the small intestine (Bonuccelli et al., 2005). In a model of A/J mice, spore ingestion with a non-capsulated strain affected immunoglobulin (Ig)A-secreting B1 cells and type 2 innate lymphoid cells (ILC2; Sahay et al., 2014), while studying the microbiota the same team demonstrated on the same model that GI anthrax infection induced a profound gut dysbiosis, breakdown of barrier function and systemic dissemination of not only *B. anthracis* vegetative bacilli, but also commensals (Lightfoot et al., 2014).

Clearly, further studies are required to understand the physiology of intestinal anthrax. The ecology of the intestinal tract is very different from that of the respiratory tract, in the sense that a third player –the microbiota- is affected along the infection. This will most probably influence the interactions between *B. anthracis*, its host and its microbiota, and the way each reacts and adapts to the encounter. Specific intestinal microbiota may protect or favor the GI anthrax by competition or synergy. Among the factors that most probably play a significant role are bacterial competition with the endogenous intestinal flora, the relative levels of O_2_ and CO_2_ and the presence of anaerobiotic zones and their consequences on germination triggering and virulence factor production. Another intrinsic characteristic of the digestive tract is that its content is subjected to a constant transit, as it is an “open tube” in contrast to the respiratory tract, which is a “dead-end tube.”

Digestive anthrax is usually considered as the main cause of infection in animals. The physiology of infection in polygastric ruminants certainly presents specificities. As the main origin of natural infection in humans is through handling of animals having died from anthrax or of their derived products, a better understanding of veterinary infection may help devise better epidemiologic control both in animals and in humans.

## Concluding Remarks

The last decade of intensive funding on bioterrorism after the 2001 bioterrorist attacks has yielded a number of advances in knowledge on anthrax pathogenesis and some novel therapeutics, but important gaps of knowledge remain.

Non-invasive imaging technologies have recently enabled real time tracking of the infection *in vivo* and have helped unravel key steps in the interactions between *B. anthracis* and its host, such as detection of new portals of spore entry (NALT, Peyer’s patches). New dynamic imaging technologies from the animal down to the microscopic level have improved our knowledge on this insidious and dreadful disease. Emerging “omics” (transcriptomic, proteomic, metabolomics) technologies will further help to elucidate additional avenues to control the infection. This integrative approach may lead to a better understanding of dynamic biological processes in the context of intact organ systems.

The main current limitation is that these technologies are restricted for use in small animals. The hope is that in the near future such powerful technologies would be applicable to larger animals that can be considered, according to some criteria, as models closer to humans. It must be also recalled that studies of *B. anthracis* infection require these technologies to be available in BSL3 (or at least in BSL2) confinement, thus requiring financial commitment and investment, together with a political will to dedicate costly equipment to infectious studies. In the meantime, data gained with the available models and tools help shape an interpretative model of what occurs in human infection. Each animal model has its limits that one should be aware of, and the intrinsic value of the data obtained in each model should be recognized, as they may help focus on key steps/events when translation to larger animals will be possible (Goossens, 2009).

An unexpected consequence of the anthrax letter attack has been the implementation of very restrictive laws and regulations to control the access to and the use of microorganisms and toxins known as the Select Agents and Toxins List (SATL) in the USA (Casadevall and Relman, 2010), and *Micro-Organismes et Toxines* (MOT) regulation in France. If these microbial threat lists have afforded some benefits for the society in term of biosecurity and biosafety, their drawbacks have also affected the scientific community, by limiting the access to the pathogen to well-funded labs and increasing the costs for the institutions housing laboratories working on biodefense. The reactions of the scientific community to the implementation of these new regulations have been very limited so far (Casadevall and Relman, 2010), but the indirect cost for the society may be important in the future by having blindly sterilized research potentials.

Crossing the epithelial barrier is not a passive phenomenon, nor is it a simple mechanical transfer of particles across a barrier. It represents a complex network of interactions between the pathogen, even for inert particles as *B. anthracis* spores, the epithelial cells and all the residing and recruited immune cells. The pathogens are confronted with “intelligent gateways” that react and adapt to the incoming danger, raising the defense machinery of the host. *B. anthracis*, in response, has either to remain silent during the crossing and exploit the natural sampling mechanisms of the host, or attempt to subvert these defenses with its toxins and maybe sacrifice itself while enabling other spores to cross the barrier more easily.

### Conflict of Interest Statement

The authors declare that the research was conducted in the absence of any commercial or financial relationships that could be construed as a potential conflict of interest.
